# From leaf out to leaves off: the effect of rising temperatures on oak's growing season length

**DOI:** 10.1093/plphys/kiag703

**Published:** 2026-09-19

**Authors:** Laura Fernández-de-Uña

**Affiliations:** Assistant Features Editor, Plant Physiology, American Society of Plant Biologists; Department of Plant Biology and Soil Sciences, Universidade de Vigo, Edificio Politécnico, As Lagoas s/n, Ourense 32004, Spain

As a result of the cyclical variability in environmental conditions throughout the year, trees in temperate and boreal latitudes adjust the phenology (ie, the timing) of their physiological and developmental processes to favorable conditions. Trees may tolerate, avoid, or escape unfavorable conditions (Vitasse et al. 2014). For example, evergreen trees tolerate winter temperatures and summer heat and drought partly due to thick cuticles covering their leaves. Trees may also avoid frost- and radiation-related damage during the winter by altering leaf chlorophyll and carotenoid concentrations, downregulating photosynthesis and upregulating photoprotection (Murphy et al. 2025). On the other hand, deciduous species escape freezing damage in leaves by remaining leafless during winter dormancy (Vitasse et al. 2014). This entails the degradation of chlorophyll and recycling of leaf nutrients (leaf senescence) and shedding leaves (leaf fall) during the autumn before freezing temperatures arrive and adjusting the timing in which new leaves are produced (budburst and leaf unfolding) to limit frost damage in the spring (Vitasse et al. 2014).

The time between leaf unfolding and leaf senescence marks the photosynthetically active period in deciduous trees (ie, the growing season). Therefore, changes in phenology not only affect tree fitness to their growing environment but also ecosystem productivity (Ren et al. 2024). Temperatures and photoperiod are key cues for leaf dormancy release and budburst, preventing frost damage to newly formed leaves (Menzel et al. 2006, Vitasse et al. 2014). Rising temperatures have therefore led to an advancement in leaf out during recent decades, increasing growing season length (Menzel et al. 2006). On the other hand, the environmental factors modulating leaf senescence, including temperature, summer drought, and daylength (Delpierre et al. 2009; Wu et al. 2022), are less universal.

Plant phenological variation is fundamentally determined by phenotypic plasticity and genetic differentiation (Valladares et al. 2014). Phenotypic plasticity represents a plant's capacity to rapidly and reversibly modify morphological, physiological, and/or phenological traits in response to short-term environmental variability, without associated genetic changes. Conversely, genetic differentiation implicates the long-term adaptation of a species or population to their native environment as a result of natural selection, in such way that plant fitness is optimized to those particular environmental conditions (Savolainen et al. 2007). The interaction between these 2 mechanisms determines species and population capacity to respond and adapt to climate change. Therefore, understanding the drivers of plant phenology is essential to assess short- and long-term responses to changing environmental conditions and improve projections under climate change scenarios.

In a recent *Plant Physiology* research paper, Zhou et al. (2026) assessed how leaf phenology (leaf unfolding, leaf senescence, and growing season length, Fig. 1a) were affected by temperatures in sessile oak (*Quercus petraea*). The authors combined 3 approaches to disentangle the effect of tree provenance and temperature on leaf phenology:

1. in situ monitoring (2005–2019) along an altitudinal gradient in the French Pyrenees (130–1630 m), where trees grew in their natural environment and phenological differences were largely due to environmental variability across sites (without neglecting provenance-level differences among the 14 studied populations);
2. a common garden, where trees from different geographic provenances were planted under the same environmental conditions and thus phenological differences were modulated by provenance-level variability among populations (2009–2018); and
3. a reciprocal transplant experiment with 5 sites along an altitudinal gradient where trees sourced from 3 different altitudes (400, 800, and 1200 m) were planted, allowing for the simultaneous assessment of provenance and environmental effects (2007–2012).

**Figure 1:**
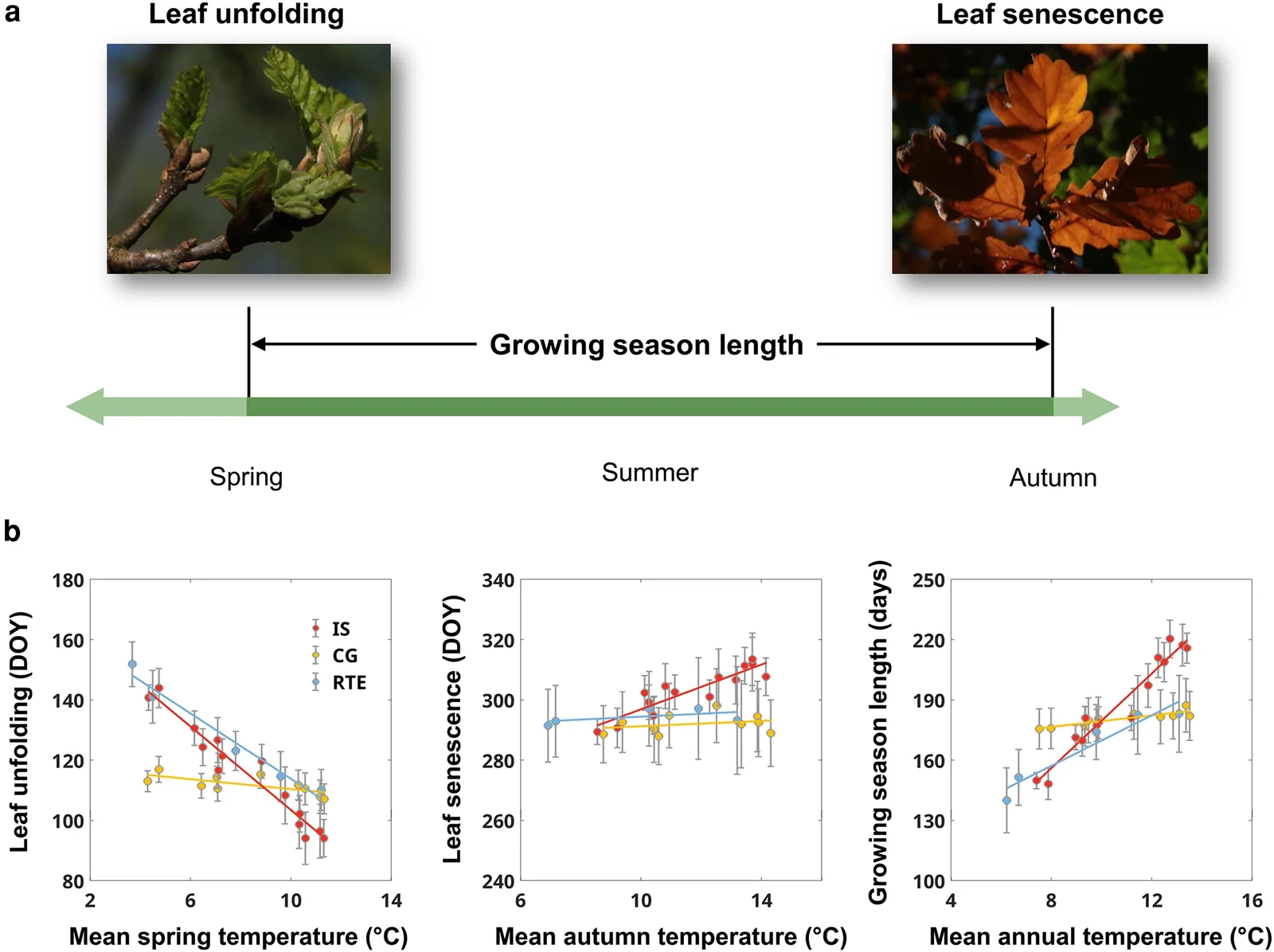
Effect of temperature on leaf phenology. (a) Phenological traits assessed (leaf unfolding, leaf senescence, and growing season length) and directionality of the change observed by Zhou et al. (2026) with increasing temperatures. (b) Response of leaf unfolding, leaf senescence, and growing season length to, respectively, mean spring, autumn, and annual temperatures (adapted from Zhou et al. 2026). CG, common garden; DOY, day of the year; IS, in situ experiment; RTE, reciprocal transplant experiment.

The authors found that increasing temperatures significantly hastened leaf unfolding, particularly in the in situ experiment where an advancement of ca. 6 days/°C was observed (Fig. 1b). Similarly, leaf unfolding was advanced with increasing growing degree days (GDD, ie, the cumulative number of degrees above 5 °C, the threshold above which plant growth occurs) and delayed with increasing chilling days (which measure the number of days with mean temperature below 5 °C). In the common garden, trees from warmer-provenance populations unfolded their leaves earlier than those from colder provenances, although this temperature effect was much weaker than in the other 2 experiments (1 d/°C). While substantial intrapopulation variability existed in leaf unfolding, population-level differences in temperature sensitivity were negligible. These results suggest that leaf unfolding is highly responsive to temperature, and more strongly influenced by phenotypic plasticity than provenance-level differentiation. This high plasticity in spring phenology may enable sessile oak to rapidly respond to climate warming, extending its growing season and maximizing carbon uptake. However, it may also increase leaf vulnerability to late-spring frosts (Vitasse et al. 2014).

Conversely, leaf senescence was less sensitive to increasing temperatures. While it was delayed in the in situ experiment by ca. 3 days/°C, no significant effect of warming or provenance was observed in the common garden or reciprocal transplant experiments (Fig. 1b), suggesting weaker temperature-related phenotypic plasticity. This was partly due to the divergent responses among trees, with both delays and advancements being observed among provenances in response to autumn temperature. Other factors, such as summer water deficits (Wu et al. 2022) and/or decreasing daylength (Delpierre et al. 2009), may have a stronger impact on leaf senescence than temperature.

Growing season length reflects changes in both leaf unfolding and senescence (Fig. 1a), and tended to be extended under warmer conditions, particularly in situ (11 days/°C) and in the reciprocal transplant experiment (6 days/°C), but rather moderately in the common garden (1 d/°C). However, as a result of the high inter-tree variability in leaf senescence, some trees showed a shortening in growing season length with increasing temperatures.

Plant phenology plasticity allows for rapid short-term responses to changes in environmental conditions, whereas genetic diversity within and among populations improves long-term resilience due to differential susceptibility to harmful conditions. Preserving genetic diversity within and among populations therefore ensures the short- and long-term adaptive capacity of forest ecosystems under a warming climate. Zhou et al. (2026) showed that phenotypic plasticity, rather than provenance-related differences, is the key driver of the response of leaf unfolding to warming in sessile oak. Conversely, the high inter-tree variability in leaf senescence response to temperature suggests that this process is affected by genotype-by-environment interactions, with provenance-level differentiation explaining a very limited proportion of the variability in autumn phenology. Future research should explore whether these patterns are maintained across species and how other environmental drivers, such as summer drought and daylength (Delpierre et al. 2009; Wu et al. 2022), interact with genetic effects in explaining leaf phenology variability, particularly in autumn.

## Recent research articles in *Plant Physiology*

- Murphy et al. (2025) showed that the phenology of photosynthesis was more sensitive to autumn warming than the phenology of primary and secondary growth.
- Deslauriers et al. (2025) found that warming and defoliation advanced budburst in balsam fir and black spruce.

## Data Availability

No new data were generated or analyzed in support of this article.
